# Generation of an X-ray nanobeam of a free-electron laser using reflective optics with speckle interferometry

**DOI:** 10.1107/S1600577520006980

**Published:** 2020-07-01

**Authors:** Takato Inoue, Satoshi Matsuyama, Jumpei Yamada, Nami Nakamura, Taito Osaka, Ichiro Inoue, Yuichi Inubushi, Kensuke Tono, Hirokatsu Yumoto, Takahisa Koyama, Haruhiko Ohashi, Makina Yabashi, Tetsuya Ishikawa, Kazuto Yamauchi

**Affiliations:** a Department of Precision Science and Technology, Graduate School of Engineering, Osaka University, 2-1 Yamada-oka, Suita, Osaka 565-0871, Japan; b RIKEN SPring-8 Center, 1-1-1 Kouto, Sayo-cho, Sayo-gun, Hyogo 679-5148, Japan; c Japan Synchrotron Radiation Research Institute, 1-1-1 Kouto, Sayo-cho, Sayo-gun, Hyogo 679-5198, Japan; d Center for Ultra-Precision Science and Technology, Graduate School of Engineering, Osaka University, 2-1 Yamada-oka, Suita, Osaka 565-0871, Japan

**Keywords:** X-ray free-electron laser, speckle interferometer, multilayer KB mirror, beam diagnosis, nano-focusing

## Abstract

Ultimate focusing of an X-ray free-electron laser (XFEL) enables the generation of ultrahigh-intensity X-ray pulses. Although sub-10 nm focusing has already been achieved using synchrotron light sources, the sub-10 nm focusing of XFEL beams remains difficult mainly because the insufficient stability of the light source hinders the evaluation of a focused beam profile. This problem is specifically disadvantageous for the Kirkpatrick–Baez (KB) mirror focusing system, in which a slight misalignment of ∼300 nrad can degrade the focused beam. In this work, an X-ray nanobeam of a free-electron laser was generated using reflective KB focusing optics combined with speckle interferometry. The speckle profiles generated by 2 nm platinum particles were systematically investigated on a single-shot basis by changing the alignment of the multilayer KB mirror system installed at the SPring-8 Angstrom Compact Free-Electron Laser, in combination with computer simulations. It was verified that the KB mirror alignments were optimized with the required accuracy, and a focused vertical beam of 5.8 nm (±1.2 nm) was achieved after optimization. The speckle interferometry reported in this study is expected to be an effective tool for optimizing the alignment of nano-focusing systems and for generating an unprecedented intensity of up to 10^22^ W cm^−2^ using XFEL sources.

## Abstract 2

Focusing of an X-ray free-electron laser enables the production of ultrahigh-intensity X-ray pulses. X-ray nanobeams of a free-electron laser were generated using reflective focusing optics combined with speckle interferometry.

## 1. Introduction   

X-ray free-electron lasers (XFELs) (Emma *et al.*, 2010 ▸; Ishikawa *et al.*, 2012 ▸; Kang *et al.*, 2017 ▸; Tschentscher *et al.*, 2017 ▸) possess unique properties, such as unprecedented peak brilliance, nearly full spatial coherence, and an ultrashort pulse duration, compared with the conventional X-ray sources. This novel light source has created new opportunities in various fields of science, such as non-destructive observation of objects based on the ‘diffraction-before-destruction’ scheme (Neutze *et al.*, 2000 ▸; Chapman *et al.*, 2011 ▸; Kimura *et al.*, 2014 ▸), and observation of molecular dynamics and ultrafast phenomena (Picón *et al.*, 2016 ▸). Furthermore, the high intensity generated by focusing the XFEL beams allows us to study the nonlinear phenomena in the X-ray region and the high-energy-density states in matter (Wehrenberg *et al.*, 2017 ▸).

To focus the XFEL beams, total-reflection Kirkpatrick–Baez (KB) mirrors (Kirkpatrick & Baez, 1948 ▸; Ice *et al.*, 2000 ▸; Yumoto *et al.*, 2013 ▸) have been widely used because of their high efficiency without chromatic aberrations and high radiation hardness accompanied by a long beam footprint. At the SPring-8 Angstrom Compact Free-Electron Laser (SACLA), Japan (Ishikawa *et al.*, 2012 ▸), a focused beam of 50 nm has been generated using the total-reflection KB mirror system (Mimura *et al.*, 2014 ▸). A peak intensity of 10^20^ W cm^−2^ was attained, which enabled the observation of various nonlinear X-ray phenomena (Tamasaku *et al.*, 2014 ▸; Yoneda *et al.*, 2014 ▸, 2015 ▸).

For higher intensities of up to 10^22^ W cm^−2^, a multilayer KB mirror system (Mimura *et al.*, 2010 ▸; Cesar *et al.*, 2017 ▸) has been developed to focus XFEL beams of less than 10 nm using a larger numerical aperture than that achieved with the total-reflection KB mirrors. To fabricate the multilayer KB mirrors with a λ/4 wavefront accuracy, an ultraprecise shape-correction technique, which involves shape-error determination with wavefront sensing (Rutishauser *et al.*, 2011 ▸; Matsuyama *et al.*, 2012 ▸; Kayser *et al.*, 2017 ▸; Inoue, Matsuyama *et al.*, 2018 ▸) and shape-correction with differential deposition at sub-nanometre accuracy (Ice *et al.*, 2000 ▸; Handa *et al.*, 2008 ▸), has been developed (Matsuyama *et al.*, 2018 ▸).

However, the evaluation of the size of the focused XFEL beam with nanometre-level accuracy remains difficult. This problem is prominent specifically in the KB focusing system, because even a small detuning angle of ∼300 nrad could broaden the focused beam size. The simple knife-edge scan method or the ptychographic method (Vila-Comamala *et al.*, 2011 ▸) are not applicable to the evaluation of focused beam sizes of less than 50 nm, mainly because of the shot-to-shot pointing fluctuation of the XFEL beam (Tono *et al.*, 2011 ▸), which differs from the nanobeam characterization for synchrotron light sources with high stability (Mimura *et al.*, 2010 ▸). Therefore, wavefront sensing techniques, such as grating interferometers, have been developed as alternatives. However, applying such techniques for the sub-10 nm focusing optics with a large numerical aperture (∼10 mrad) is generally difficult, because various systematic errors could arise because of the misalignment of the interferometer or imperfections in system devices such as gratings and X-ray cameras. Therefore, the exigency of direct evaluation of the nanobeam profile on a single-shot basis demands a new method.

Accordingly, we present an approach in this study that involves characterising a beam size and profile using speckles from a phase object located around the focus, because the size of the speckle in coherent X-ray scattering from fine particles inversely changes with the broadening of a focused beam (Sikorski *et al.*, 2015 ▸). A major advantage of this method is the direct determination of the beam size in two dimensions (2-D) with a single shot at the focus, which is the same as that required for generating the XFEL nanobeam.

In this paper, we present the generation of an XFEL nanobeam by optimizing the KB mirror alignment using speckles. Computer simulations indicated excellent correlation between the beam profile and the alignment with extremely high accuracy. In addition, we provide experimental evidence that an XFEL of less than 10 nm was attained at SACLA.

## 2. Theory of beam diagnosis using speckles by coherent scattering   

By assuming coherent light illumination, an incident beam with complex amplitude of *p*(*x*, *y*) and a sample with complex transmission function of *o*(*x*, *y*) create an exit wave with complex amplitude of ψ(*x*, *y*) immediately after the sample, which can be expressed as
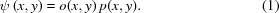
The complex amplitude *A*(*u*, *v*) in the observation plane, *i.e.* the speckle field, relates to ψ(*x*, *y*) by Fresnel propagation as follows,
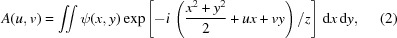
where (*u* , *v*) is a scattering vector and *z* is the distance between the sample and the observation planes. The objective of this study is to calculate the autocorrelation function γ_*A*_ of the speckle field *A*(*u* , *v*) at the two points (*u*_1_, *v*_1_) and (*u*_2_, *v*_2_), as follows,
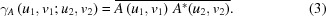
A simple equation can be obtained as follows (Goodman, 2007 ▸),
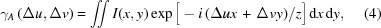
where Δ*u* = *u*_1_ − *u*_2_, Δ*v* = *v*_1_ − *v*_2_, and *I*(*x*, *y*) is the intensity of ψ(*x*, *y*). Therefore, the autocorrelation function of the complex amplitude of the speckle is represented by a simple 2-D Fourier transform of the intensity distribution of the exit wave.

The sample is assumed to be an object consisting of nanometre-size particles that are spread uniformly on a thin membrane with a thickness of a few micrometres. Thus, the absorption over the entire surface of the sample can be assumed to be uniform, and *I*(*x*, *y*) is thus equal to |*p*(*x*, *y*)|^2^.

Only the intensity distribution in the speckle pattern can be measured; thus, the autocorrelation function γ_*I*_(Δ*u*, Δ*v*) for the speckle pattern intensity can be expressed as follows,
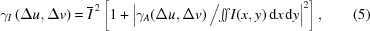
where 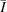 is the average intensity of the speckle field. Because the autocorrelation of a speckle pattern retains the information about the beam size, the profile can be deduced from a speckle pattern. When Δ*u* = Δ*v* = 0, the right-hand side of equation (5) contains an additional term 1/|*A*(*u*, *v*)|^2^, where |*A*(*u*, *v*)|^2^ is the average number of detected photons per pulse at (*u*, *v*), attributed to the photon statistics (Singer *et al.*, 2014 ▸; Inoue, Hara *et al.*, 2018 ▸).

For simplicity, when *I*(*x*, *y*) follows a Gaussian distribution, it can be expressed as
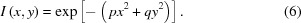
Then, γ_*I*_(Δ*u*, Δ*v*) is described as follows,
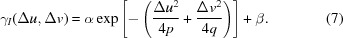
where α are β constants. Equations (6) and (7) show that the beam size is inversely proportional to the average speckle size in γ_*I*_.

## 3. Computer simulation   

### 3.1. Relationship between mirror misalignments and beam profile   

When aligning the KB mirrors, high accuracy is required for the perpendicularity between the mirrors Δχ, incident angles Δθ, and the relative position of the two mirrors along the optical axis Δ*d* (*i.e.* astigmatism). We calculated the influence on the beam profile near the focus caused by misalignments, as well as tolerances. To simplify the calculation, we used a lens model [refer to Matsuyama *et al.* (2018 ▸) for more details on parameters], which consists of a pair of one-dimensional lenses with a numerical aperture of 0.01 and focal length of 70 mm that are orthogonally arranged with each other. The wavefields around the focus were calculated by changing the distance from the focus Δ*z* (*i.e.* defocus), based on the Fresnel diffraction at an X-ray energy of 9.1 keV.

**Figure 1:**
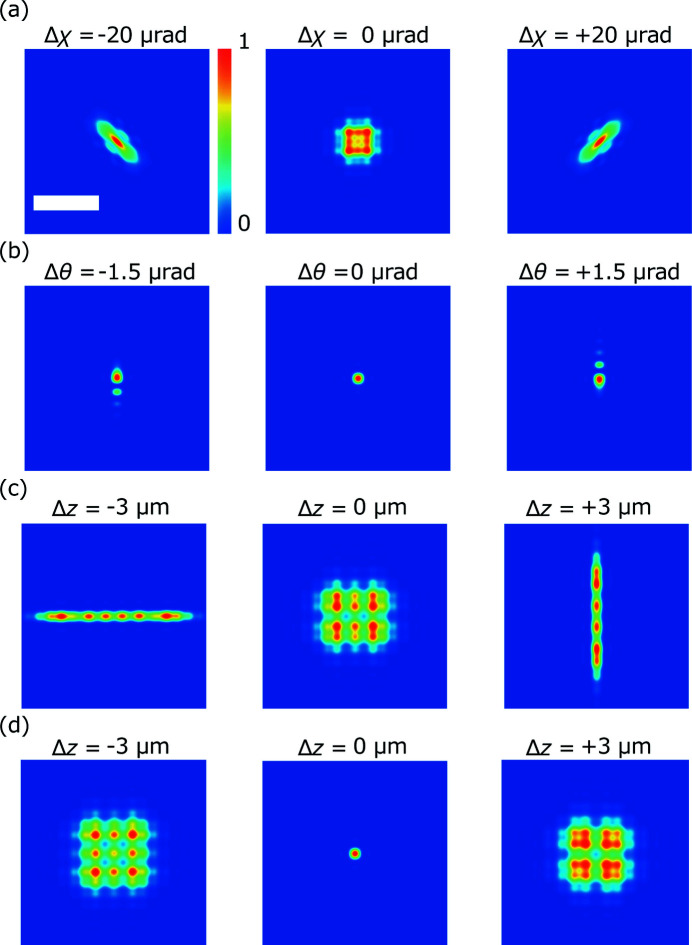
Calculated beam profiles near the focus. Intensity profiles at (*a*) the defocus position Δ*z* = 1.6 µm with the perpendicularity errors of Δχ = ±20 µrad and 0 µrad, and (*b*) focus with the incident angle errors of Δθ = ±1.5 µrad and 0 µrad. Intensity profiles at Δ*z* = ±3 µm and 0 µm (*c*) with astigmatism Δ*d* = 6 µm and (*d*) without astigmatism. Scale bar: 50 nm.

Fig. 1 ▸ shows the calculated results for intensity profiles around the focus after introducing the misalignments of perpendicularity, astigmatism and incident angle. They were calculated for the following conditions: perpendicularity errors of Δχ = ±20 µrad, incident angle errors of Δθ = ±1.5 µrad, and astigmatism of Δ*d* = 6 µm. For reference, we performed similar calculations without errors.

The calculation results yielded the following conclusions. The perpendicularity errors distorted the beam to a tilted ellipse, and astigmatism caused a mismatch of the focus positions in the vertical and horizontal directions. The incident angle errors generated satellite peaks on one side. The tolerances for the misalignments were estimated based on the Strehl ratio (Strehl, 1895 ▸, 1902 ▸) and Marechal criterion, as shown in Table 1 ▸.

### 3.2. Relationship between speckle profile and misalignments   

**Figure 2:**
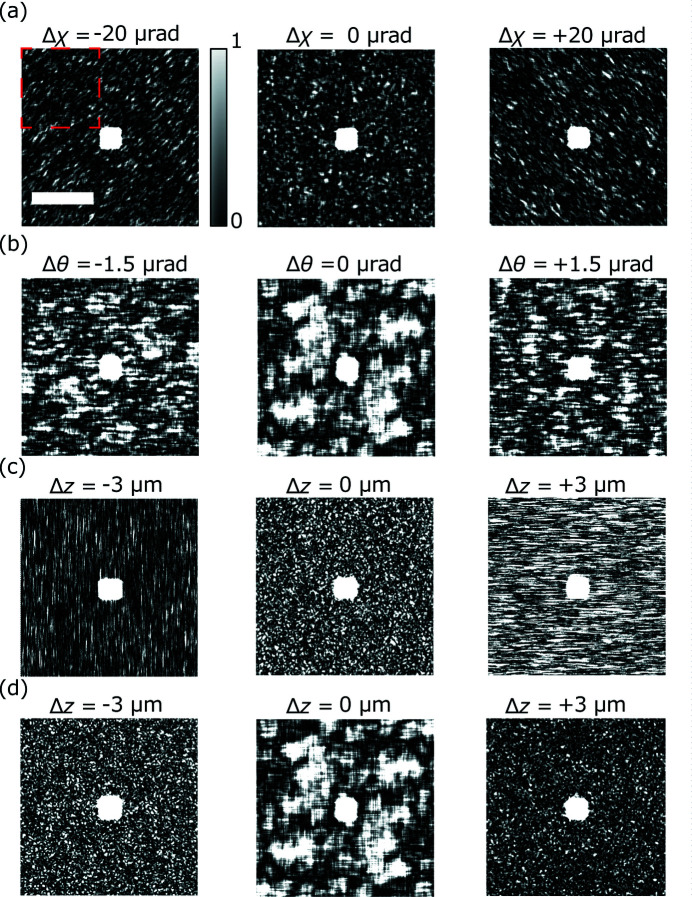
Calculated speckle patterns of nanoparticles using the complex probe function corresponding to Figs. 1(*a*)–1(*d*) ▸. Scale bar: 0.5 nm^−1^. The center of each figure corresponds to bright field. The area enclosed by the red dotted line was used to calculate the autocorrelation function.

The speckle patterns and the speckle profile were calculated using Fourier transformation and autocorrelation. Fig. 2 ▸ shows the calculated speckle patterns of the randomly distributed nanoparticles shown in Table 2 ▸, based on the complex probe function calculated for Fig. 1 ▸. The speckle profiles were determined by calculating the autocorrelation function of these speckle patterns.

**Figure 3:**
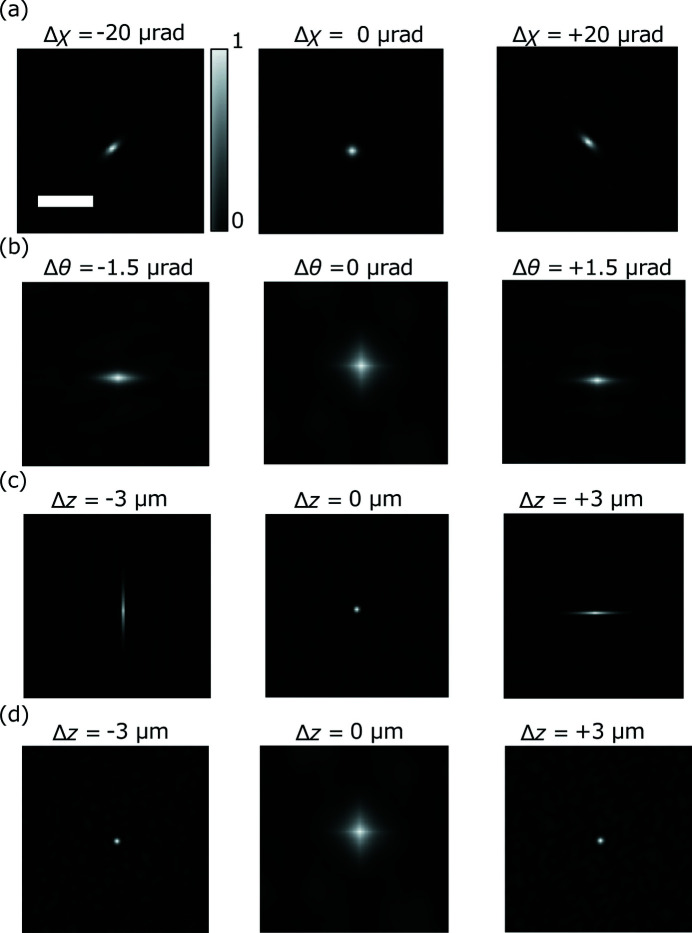
Autocorrelation function of the speckle patterns corresponding to Figs. 2(*a*)–2(*d*) ▸. Scale bar: 0.15 nm^−1^.

**Figure 4:**
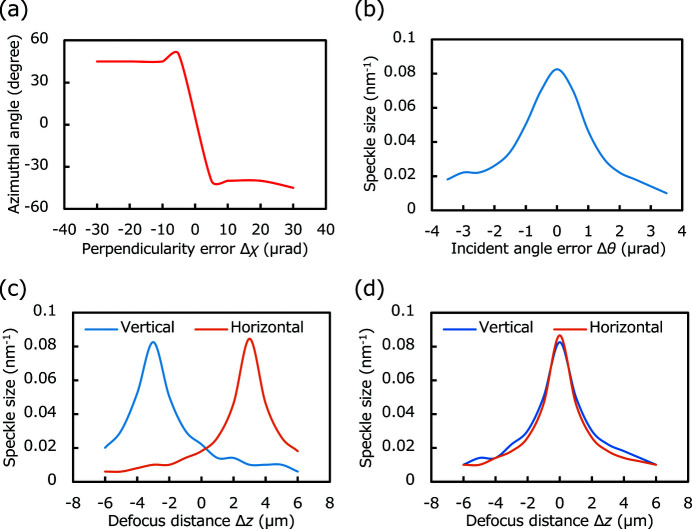
Analysis results of the speckles. (*a*) Azimuthal angle of the speckle profile versus perpendicularity error. (*b*) Speckle size versus incident angle error. Speckle sizes in the vertical and horizontal directions versus sample position along the optical axis (*c*) with astigmatism Δ*d* = 6 µm and (*d*) without astigmatism.

Fig. 3(*a*) ▸ shows that the misalignment of perpendicularity deforms the speckle profile into a tilted ellipse. Fig. 4(*a*) ▸ reveals that the azimuth of the average speckle is highly sensitive to the perpendicularity error Δχ with the required accuracy (±9 µrad). Figs. 3(*b*) ▸ and 4(*b*) ▸ reveal that any error in the incident angle Δθ reduces the speckle size at the focal position, and the speckle size increases when the incident angle is optimized. The optimum incident angle can be determined with the required accuracy (±0.9 µrad). Figs. 3(*c*) and 3(*d*) ▸ show that the speckles were one-dimensionally shrunk by the defocus caused by astigmatism Δ*d*. The two peaks in Fig. 4(*c*) ▸ clearly indicate that the focus positions in the vertical and horizontal directions deviated with a separation distance of 6 µm, which is equal to the introduced value of astigmatism; further, the two peaks can be resolved with the required accuracy (±1 µm). Based on these findings, an alignment procedure for the KB mirror was established.

## 4. Experiment   

**Figure 5:**
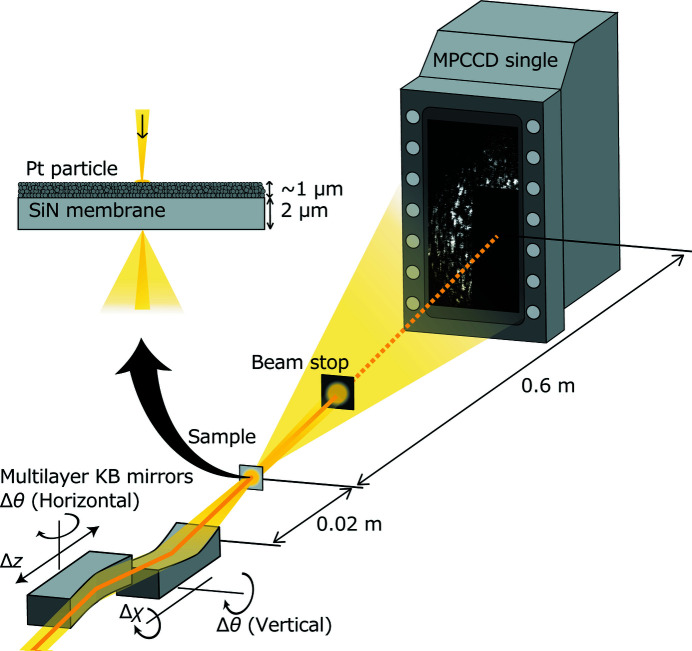
Schematic of the experimental setup. Platinum particles that were dispersed on a SiN membrane were irradiated by focused XFELs, and the scattered light was recorded using an MPCCD. A beam stop was installed between the sample and the MPCCD.

The experiment was conducted at SACLA BL3 under the following conditions: photon energy of 9.1 keV, pulse energy of approximately 11 µJ at focus, and an estimated pulse duration of 7 fs (Inubushi *et al.*, 2012 ▸ 2017 ▸). Based on previous studies (Kim *et al.*, 2015 ▸), the power density of the XFEL beams on our mirrors was much lower than the damage threshold. The sub-10 nm focusing system at experimental hutches 4 and 5 was used to focus the XFELs. A single-type multi-port charge-coupled device (MPCCD) (Kameshima *et al.*, 2014 ▸) detector was placed to obtain the speckle patterns at 600 mm downstream of the focus (Fig. 5 ▸).

To avoid radiation damage to the MPCCD, a 1 mm-thick Ta beam stop (BS) was placed between the focus and the MPCCD. To measure bright speckles, the particle size must be smaller than the diffraction limit of the optical system. A slurry composed of platinum particles of 2 nm diameter and an organic solvent (Tanaka Kikinzoku Kogyo Co. Ltd) was spread on a 2 µm-thick SiN membrane (NTT Advanced Technology Co.) using a micropipette. The thickness of the particle layer was measured using a laser-type CMM (Mitaka Kohki Co. Ltd, NH-3SP) by dropping a 40 µL mixture to be 1 µm.

The sample was irradiated by a single-pulse focused XFEL to obtain the speckle pattern. A uniform region (area = 500 × 200 pixels) from a single-shot speckle pattern was used to calculate the 2-D autocorrelation function, and the speckle size (FWHM) in the vertical and horizontal directions was evaluated from the line profile of the fitted autocorrelation functions. Because of Poisson noise and charge sharing, fitting was performed except for the center and a position 1 pixel away from the center.

## 5. Results and discussion   

**Figure 6:**
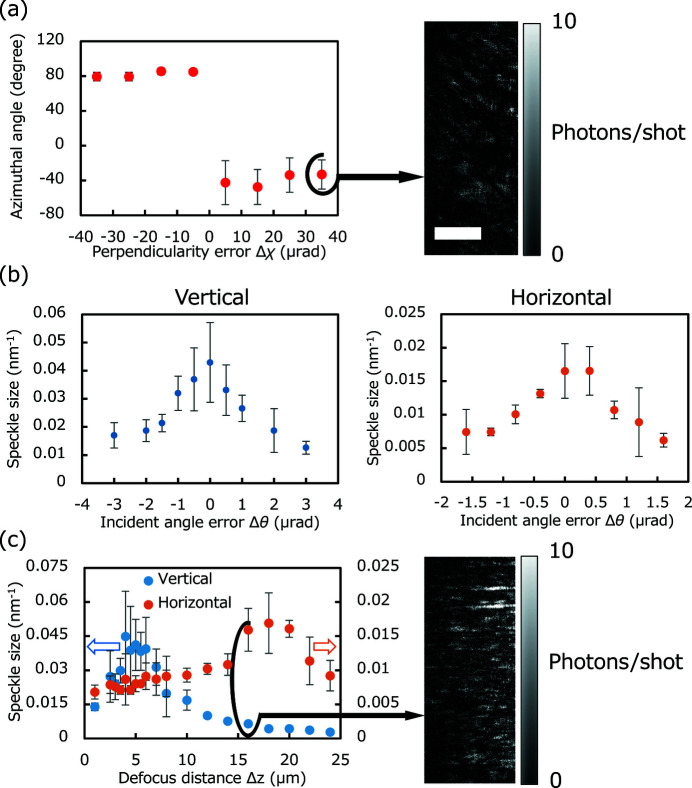
Analysis results of experimental speckle patterns. Each point consists of three to ten images on average, and the error bar represents the standard deviation. (*a*) Azimuthal angle of speckle versus perpendicularity error Δχ. The image on the right represents the corresponding speckle pattern obtained at the error of 35 µrad (FWHM of the average speckle of 0.3 nm^−1^ × 0.12 nm^−1^). Scale bar: 0.06 nm^−1^. (*b*) Speckle size versus incident angle error Δθ in the vertical and horizontal directions. (*c*) Speckle size in the vertical and horizontal directions under astigmatism Δ*d* versus sample position along the optical axis. The image on the right represents the speckle pattern obtained at the relative distance of 16 µm (FWHM of the average speckle of 0.3 nm^−1^ × 0.12 nm^−1^). Scale bar: 0.06 nm^−1^.

Fig. 6(*a*) ▸ shows the azimuthal angle of the average speckle based on the perpendicularity error Δχ with a pitch of 10 µrad. A large change in azimuthal angle can be seen around the optimized angle, which is easily detected with the required accuracy. Fig. 6(*b*) ▸ shows the average speckle size variation with the incident angle error, in which the sample position was always optimized along the optical axis to remove the defocus effect by changing the incident angle Δθ. These plots reveal that the optimized incident angles can be determined with an accuracy of approximately 0.5 µrad in both directions, which is very close to the required accuracy. The sensitivity in the horizontal direction was slightly low. This is because of the wavefront aberration caused by the horizontal focusing mirror. Fig. 6(*c*) ▸ portrays the relationship between the average speckle size and the defocus distance. A mismatch between the positions of the horizontal and vertical peaks was observed, which indicates the existence of astigmatism Δ*d*. The detection accuracy of position difference of the two peaks is approximately ±1.0 µm, which is comparable with the required accuracy.

**Figure 7:**
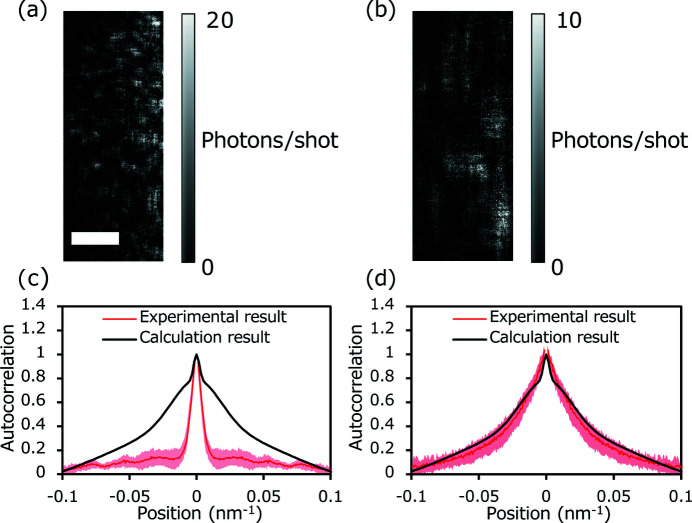
Comparison of speckle intensity patterns (*a*) before and (*b*) after adjustment. Scale bar: 0.06 nm^−1^. Vertical line profiles of the autocorrelation function (*c*) before and (*d*) after adjustment. Red lines represent the experimental results averaged over (*c*) 10 speckle patterns and (*d*) 17 speckle patterns. Areas shaded in pink represent the standard deviation. Black lines represent the calculated result at a perfect alignment condition.

We adjusted the mirror alignment using the knife-edge scan method and a grating interferometer, and acquired an image [Fig. 7(*a*) ▸]. Based on these observations, all the alignments were optimally adjusted using this beam diagnosis method. Fig. 7(*b*) ▸ displays the typical speckle patterns obtained after the alignment adjustments based on the speckle shape. The adjustment maximized the speckle size, which indicates that the mirror alignment was successfully optimized. It appeared that the speckle size in the vertical direction was larger than that in the horizontal direction, as presented in Fig. 7(*b*) ▸. This was mainly because the wavefront aberration in the horizontal direction, 0.893 rad (r.m.s.), was larger than that in the vertical direction, 0.45 rad (r.m.s.), and the incident angle of the horizontal focusing mirror was slightly degraded by temperature change (see the supporting information for details).

The speckles that were finally observed after the optimization were required to be compared with the calculated speckles, which were evaluated from the wavefront aberration of the KB mirror simultaneously measured using a grating interferometer and from a simulation model having the same numerical aperture as the experimental parameter. The vertical cross-section profiles of the autocorrelation function, represented by the red line and pink shaded area in Fig. 7(*d*) ▸, were compared with their calculation counterparts. This figure illustrates that the experimental and calculated profiles are in good agreement, indicating that the beam diagnosis method is highly effective. Finally, the beam FWHM was evaluated as 5.8 ± 1.2 nm. The uncertainties in the speckle and beam sizes were because of the insufficient calculation area to analyze the speckle size. To determine the speckle and beam sizes accurately, it is necessary to prepare particles smaller than 2 nm and expand the area in the far field that can be used for speckle calculations.

We successfully demonstrated that speckle interferometry is highly advantageous for evaluating the size and profile of the nanobeam and systematically optimizing the KB mirror alignments. The beam size information is crucial for accurately determining the beam intensity, which provides a robust basis for nonlinear X-ray optics. Additionally, this method is applicable to nano-focusing systems for synchrotron light sources. Furthermore, this method can be extended to evaluate a 1 nm focus using smaller scatterer particles or atoms.

## Figures and Tables

**Table 1:** Estimated tolerances for each misalignment of the KB mirror

	Horizontal	Vertical
Perpendicularity Δχ	±9 µrad	
Incident angle Δθ	±350 nrad	±900 nrad
Astigmatism Δ*d*	±1.0 µm	

**Table 2:** Parameters of nanoparticles used for the speckle simulation

Size	0.5 nm
Phase shift	λ/1000
Number	5000000
Area	500 × 500 nm
